# Surgical anatomy of the right hepatic artery in Rouviere’s sulcus evaluated by preoperative multidetector-row CT images

**DOI:** 10.1186/s12893-016-0155-0

**Published:** 2016-06-08

**Authors:** Shuichi Aoki, Masamichi Mizuma, Hiroki Hayashi, Kei Nakagawa, Takanori Morikawa, Fuyuhiko Motoi, Takeshi Naitoh, Shinichi Egawa, Michiaki Unno

**Affiliations:** Department of Surgery, Tohoku University Graduate School of Medicine, Aobaku, Sendai, Japan; Division of International Cooperation for Disaster Medicine, Tohoku University, 1-1 Seiryomachi, Aobaku, Sendai, 980-8574 Japan

**Keywords:** Right hepatic arteries, Cholangiocarcinoma, Postoperative complications

## Abstract

**Background:**

Lymph node dissection in Rouviere’s sulcus (RS) is essential during left-sided hepatectomy and caudate lobectomy for hilar cholangiocarcinoma. However, the small segmental or subsegmental arteries (SA/SSA) are often encountered in RS and must be preserved to prevent critical complications, such as liver infarction or liver failure. The aim of this study is to elucidate the anatomy of SA/SSA around RS, which should be understood preoperatively.

**Methods:**

Between January 2008 and April 2013 from a total of 124 consecutive patients with hilar cholangiocarcinoma, preoperative multidetector-row computed tomography (MDCT) images were obtained at our institution and evaluated. The bifurcation patterns of the SA/SSA, the courses of the posterior SA/SSA and the bifurcation site of the SA/SSA were investigated using MDCT images.

**Results:**

The typical form, in which right hepatic artery (RHA) bifurcated into the anterior (Aant) and posterior (Apost) hepatic artery and thereafter, Aant/Apost bifurcated into the SA and SSA, was observed in 75 patients (60.5 %). On the other hand, the atypical forms, in which the SA/SSA were independently branched off from RHA before the main bifurcation of the Aant and Apost, were observed in 43 patients (34.7 %). The prior branched arteries supplied the whole or ventral area of segment VI (A6 or A6a) in 11 patients (8.9 %), which was most commonly observed in the atypical form. 15 patients (34.9 %) of the 43 patients with atypical form had partially supraportal posterior branches, that showed early-bifurcated posterior SA/SAA following supraportal course, while the other posterior SA/SSA followed infraportal course. The SA/SSA were extrahepatically bifurcated in 82 patients (66.1 %), comprised of all 43 atypical form and 39 of typical form, while the SA/SSA were intrahepatically bifurcated in remaining 36 patients of typical forms (29.0 %).

**Conclusion:**

The extrahepatic bifurcation of the SA/SSA from RHA was relatively common. The early-bifurcated SA/SSA was often observed (34.7 % of total cohort) and, in 34.8 % of those atypical forms, posterior SA/SSA from RHA followed a supraportal course. The detailed preoperative knowledge of the anatomy, including SA/SSA, is crucial for left-sided hepatectomy for hilar cholangiocarcinoma.

## Background

Left-sided hepatectomy with caudate lobectomy is ordinarily applied for patients with Bismuth type IIIb hilar cholangiocarcinoma. In recent years, even if a Bismuth type IIIb tumor extends to the right-side hilum involving the right hepatic artery (RHA), long-term survival after left hepatectomy or trisectionectomy with concomitant arterial resection and reconstruction has been reported [1–3]. However, this surgical technique is still extremely difficult and has a high risk for postoperative complications. To understand thoroughly the surgical anatomy around Rouviere’s sulcus (RS) is pivotal for lymph node dissection and arterial reconstruction in left-sided hepatectomy for hilar cholangiocarcinoma.

In RS, located at the boundary between the caudate lobe and the right hepatic lobe, the branches from the right hepatic artery, right portal vein and right hepatic bile duct run into the liver parenchyma. In general, RHA is bifurcated from the proper hepatic artery (primary bifurcation) and, thereafter, bifurcates into the anterior (Aant) and posterior (Apost) hepatic artery immediately before entering into the liver parenchyma (secondary bifurcation). Then, segmental (SA) or subsegmental arteries (SSA) from Aant or Apost are bifurcated within the liver parenchyma (tertiary bifurcation). However, SA or SSA is often atypically branched off from the RHA. In addition, the posterior SA or SSA occasionally run cranially to the right portal vein (RPV) (supraportal course), although Apost typically runs caudally to RPV (infraportal course) [4]. Thus, the branching form of the arteries around RS is complicated. The preoperative understanding of anatomical variation of the SA/SSA branches and course variations of the arterial posterior branches around RS, especially the supraportal/infraportal posterior SA/SSA, is crucial in left-sided hepatectomy for hilar cholangiocarcinoma in order to avoid critical surgical complications, such as intraoperative arterial injury, hepatic infarction and hepatic failure. However, there have been few reports that provide a detailed discussion about the anatomical variation of SA/SSA in RS from the standpoint of surgical resection.

The objective of this study is to elucidate the bifurcation patterns of the SA/SSA using multidetector-row computed tomography (MDCT). We clarified the incidence of early-bifurcated SA/SSA around RS, the variations in the courses they followed. The findings of this study should be helpful for left-sided hepatectomy for hilar cholangiocarcinoma.

## Methods

### Patients

Between January 2008 and April 2013, 124 consecutive patients with hilar cholangiocarcinoma who underwent preoperative MDCT and received surgical resection at our institution were examined. The bifurcation pattern and the course of SA/SSA from RHA around RS were evaluated using preoperative MDCT. This study was approved by the institutional review board of Tohoku University, which waived the need for consent because this study was a retrospective study using enhanced CT images obtained during usual clinical examinations under written consent from all of the patients.

### MDCT imaging

MDCT is the gold standard for imaging the anatomy of the hilar vessels and assessment of the cancer progression. The 64-row MDCT (Toshiba) was performed in all patients except for those allergic to iodine contrast medium prior to the biliary drainage. In cases in which the patients had already received biliary intervention at another institution, MDCT imaging was evaluated at our institution immediately after referral. Images were taken prior to injection and at 20 s (early arterial phase); 40 s (late arterial phase); 70 s (portal phase); and 120 s (delayed phase) after injection of a nonionic contrast agent (300 mg/ml) at a rate of 4 ml/h. In addition to axial images, multi planar reformation (MPR) images were routinely obtained using OsiriX® medical imaging software (open source software, http://www.osirix-viewer.com/). Three-dimensional (3D) images of the arteries and portal vein were prepared by a volume-rendering method to improve understanding of vascular relationships.

### Bifurcation pattern of the SA/SSA from RHA

**Fig. 1:**
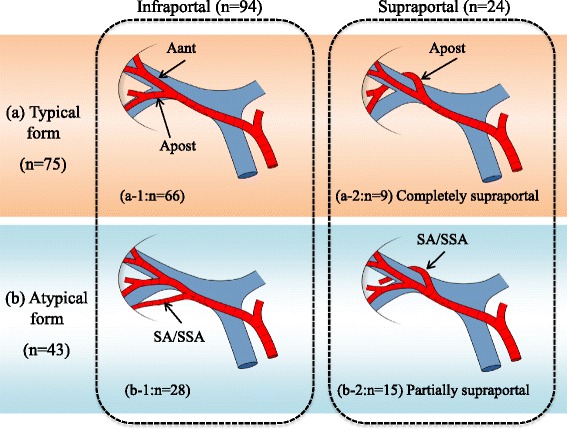
Arterial anatomy of SA/SSA from RHA around the RS. **a** typical form: RHA bifurcates into Aant and Apost, and then ramifies into the SA/SSA as a tertiary bifurcation. **b** atypical form: SA/SSA branched off independently before the main bifurcation of Aant and Apost. (*a-1*/*b-1*) infraportal course: Apost runs caudally to RPV. (*a-2*/*b-2*) supraportal course: Apost, including posterior SA/SSA, run cranially to RPV. The atypical form was observed in 43 cases. 15 cases of atypical form showed prior branched posterior SA/SSA following a supraportal type, while the other posterior branches were the infraportal type, defined as “partially supraportal type” (*b-2*)

**Fig. 2:**
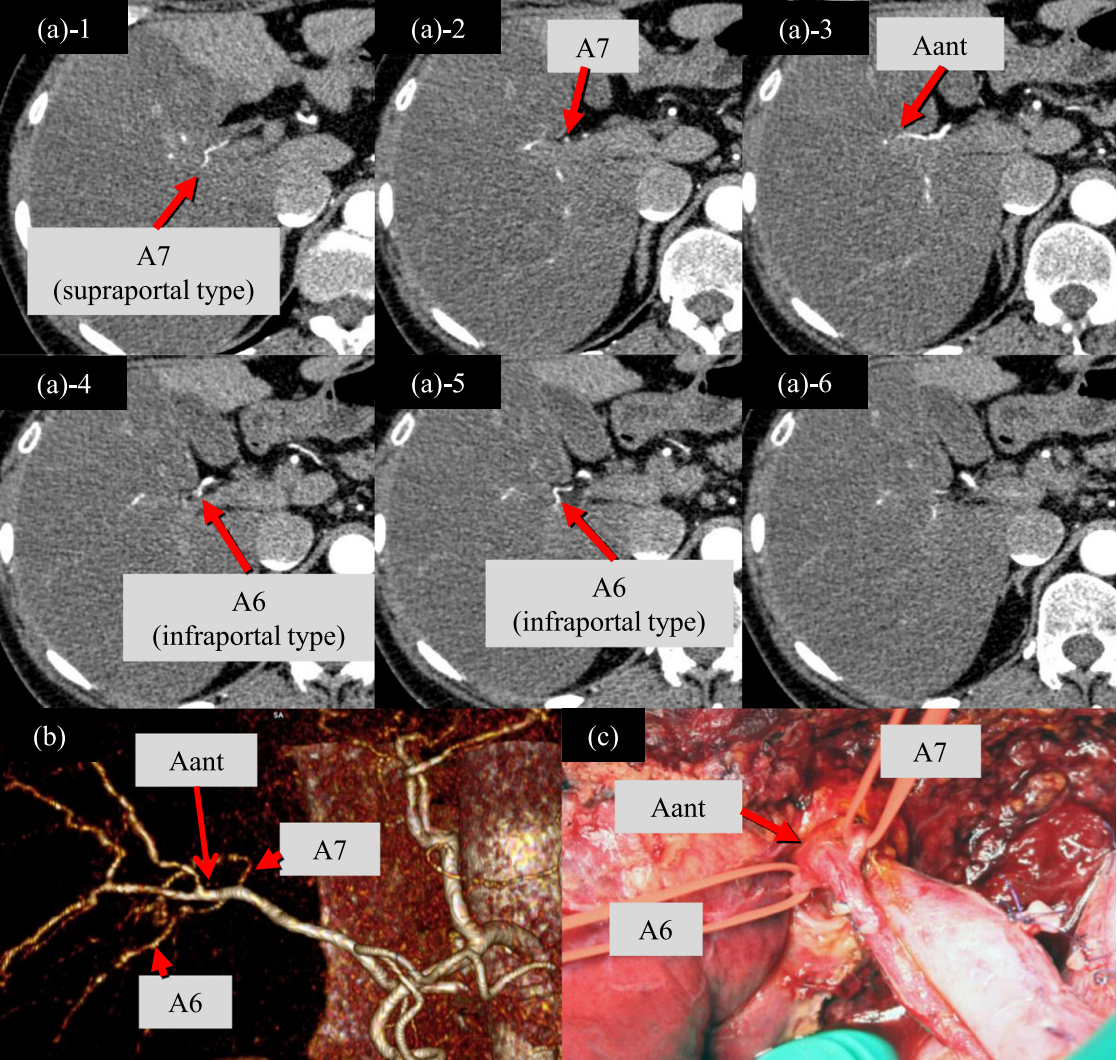
A representative case of the partially supraportal type. **a** Transverse MDCT images in early arterial phase and **b** three-dimensional arteriogram obtained in a 57-year-old woman with hilar cholangiocarcinoma. **c** Intraoperative photograph after left hepatectomy and caudate lobectomy with hilar lymph node dissection. RHA branched off A7 of the supraportal type before the bifurcation of A6 of the infraportal type and Aant. A6 and 7 supplied segment VI and VII, respectively

RHA typically bifurcates into Aant and Apost and, thereafter, Aant/Apost bifurcates into the SA and SSA, defined as the typical form. SA/SSA, such as the artery to segment VI (A6) or the artery to the ventral area of VI (A6a), often branch off separately before the main bifurcation into the Aant and Apost, classified as the atypical form (Figs. 1 and 2). The bifurcation patterns of the SA/SSA from RHA, comprised of typical and atypical forms, were evaluated. Moreover, the course patterns of the posterior SA/SSA around the RPV, namely supraportal or infraportal [4], were investigated.

**Fig. 3:**
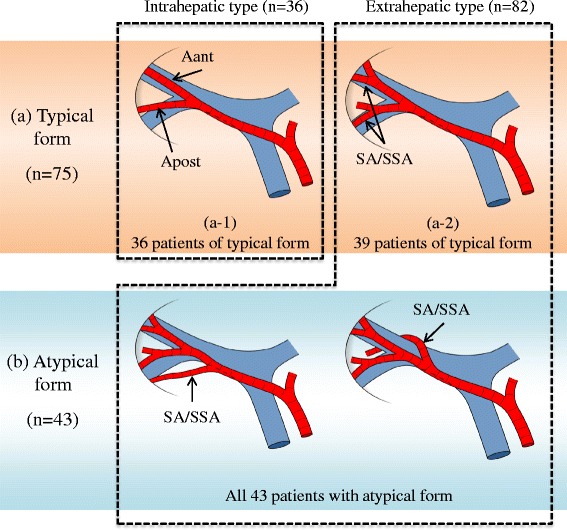
Bifurcation site of the SA/SSA (intrahepatic/extrahepatic type). **a** typical form, **b** atypical form. Intrahepatic type: The SA/SSA branches were intrahepatically bifurcated from Aant or Apost in 36 patients with the typical form (29.0 % of total cohort)*(a-1*). Extrahepatic type: Extrahepatic bifurcation of the SA/SSA branches was revealed in 82 patients (66.1 % of total cohort), consisting of 39 with the typical form (*a-2*) and all 43 with the atypical form (*b*)

**Fig. 4:**
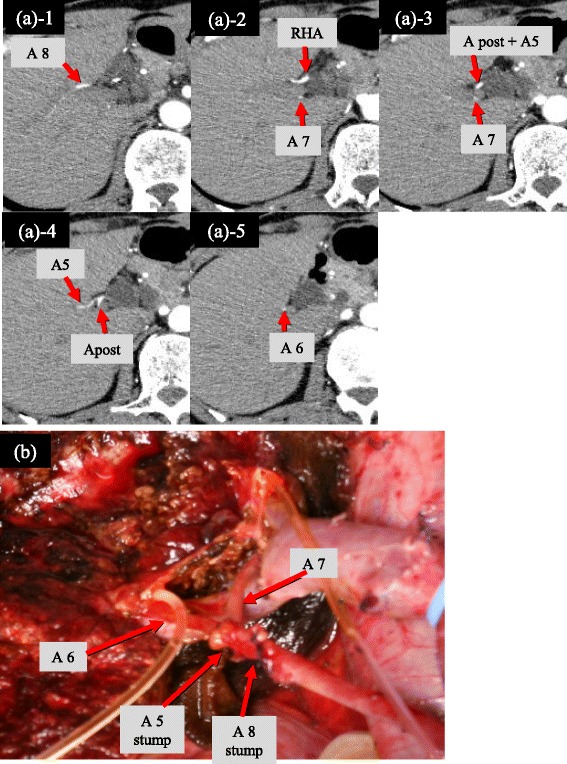
A representative case of the extrahepatic type. **a** Transverse MDCT images in early arterial phase obtained in a 57-year-old man with hilar cholangiocarcinoma. RHA bifurcated into A8 and Apost + A5 (common trunk of Apost and A5), thereafter Apost + A5 bifurcated into Apost and A5. A8 and A5 extrahepatically branched off from RHA. A8 and 5 supply segment VIII and V, respectively. **b** Intraoperative photograph after left trisectionectomy with caudate lobectomy and hilar lymph node dissection. Stumps of A5 and A8 are shown. During hilar lymph node dissection, A5 and A8 were exposed and cut

To examine whether SA/SSA bifurcated inside or outside the liver, the cohort was separated into the two groups, “extrahepatic type” and “intrahepatic type” (Figs. 3 and 4). “Extrahepatic type” was defined as a bifurcation of the SA/SSA that showed no direct contact with the hepatic parenchyma in MDCT. On the other hand, contact between the bifurcation and the hepatic parenchyma or the bifurcation inside the hepatic parenchyma was designated as the “intrahepatic type”. The frequency and anatomical characteristics of the extrahepatic and intrahepatic types were investigated.

Permission was granted by the patients for the publication of Figs. 2 and 4.

## Results

### Patient characteristics and surgical procedures

Of 124 patients (median age: 69 y.o.), 80 men and 44 women received surgical resection. Left hepatectomy, left trisectionectomy, right hepatectomy and right trisectionectomy with caudate lobectomy were performed in 50 patients (40.3 %), 9 patients (7.3 %), 60 patients (48.3 %) and 3 patients (2.3 %), respectively (Table 1). 2 patients (1.6 %) underwent biliary resection with no hepatectomy. Patients with concomitant resection and reconstruction of the portal vein and the hepatic artery were 34 (27.4 %) and 6 (4.8 %), respectively. Hepatectomy combined with pancreaticoduodenectomy was done in 22 patients (17.7 %).

**Table 1:** Patients characteristics and surgical procedures

Gender	Male		80
	Female		44
Age	Median		69
	Range		41–82
Surgical procedure	Hepatectomy^a^		
		S1,2,3,4,5,8,	9
		S1,2,3,4,	50
		S1,5,6,7,8,	60
		S1,4,5,6,7,8,	3
		Bile duct resection	2
	Other combined resection		
		Portal vein reconstruction	34
		Hepatic artery reconstruction	6
		Pancreaticoduodenectomy	22

^a^Resection area of the liver is described as Couinaud’s hepatic segment(s)

### Bifurcation pattern of RHA (typical or atypical form)

The typical form was observed in 75 patients (60.5 %) (Table 2 and Fig. 1). On the other hand, the atypical form was seen in 43 patients (34.7 %), 24 patients (19.4 %) of whom showed SA/SSA branching off separately before the bifurcation of the Aant and Apost. These separately branched arteries supplied the whole or ventral area of segment VI (A6 or A6a) in 11 patients (8.9 %), which was most commonly observed in the atypical type. Trifurcation of RHA was revealed in 18 patients (14.5 %), and indicated Aant + A6 + A7 in 9 patients (7.3 %). One patient (0.8 %) showed a RHA that diverged into four branches, designated as A6 + A7 + A8a (arteries to the ventral region of segment VIII) + A8c (arterial branches to the posterior region of segment VIII). The bifurcation pattern could not be identified in six patients (4.8 %) due to tumor infiltration.

**Table 2:** The bifurcation pattern of SA/SSA from RHA

Typical form			75 (60.5 %)		
Atypical form			43 (34.7 %)		
	SA/SSA separately branched off before main bifurcation of Aant and Apost			24 (19.4 %)	
		A5			2
		A6 or A6a			11
		A7			4
		A8 or A8a or A8c			7
	Trifurcarion of RHA			18 (14.5 %)	
		A5 + A8 + Apost			4
		A8a + A8c + Apost			5
		Aant + A6 + A7			9
	Simultaneous ramification of RHA to four branches			1 (0.8 %)	
		A6 + A7 + A8a + A8c			1
Unclear form (because of cancer invasion)			6 (4.8 %)		

*SA/SSA* segmental or subsegmental arteries, *RHA* right hepatic artery, *Aant and Apost* anterior and posterior hepatic artery, *SA/SSA* segmental or subsegmental arteries, *A5, 6, 7 and 8* segmental artery supplying segment V, VI, VII and VIII, respectively, *A6a, A8a and A8c* subsegmental artery suppling ventral segment VI, ventral segment VIII and dorsal segment VIII, respectively

### The course of the posterior SA/SSA in relation to RPV

The posterior segmental arteries including their branches followed an infraportal course or a supraportal course in 94 patients (75.8 %) or 24 patients (19.4 %), respectively (Table 3). Of 24 patients with supraportal courses, 9 patients (7.3 %) showed a completely supraportal course without any infraportal branches, while 15 patients (12.1 %) showed a partially supraportal course with infraportal branches (Fig. 1). Of the 15 partially supraportal cases, 4 patients (3.2 %) had infraportal A6 and supraportal A7 (Fig. 2). In the remaining 11 patients (8.9 %), A6a followed an infraportal course and the other posterior segmental arteries (A6bc7) followed a supraportal course. All of the partially supraportal cases, in whom the supraportal posterior branches were early bifurcated from the RHA, were classified as the atypical form. On the other hand, 15 cases (34.9 %) of the 43 atypical form cases showed early-bifurcated posterior SA/SAA with partially supraportal course (Fig. 1).

**Table 3:** The courses of the posterior SA/SSA in relation to RPV

Infraportal form			94 (75.8 %)		
Supraportal form			24 (19.4 %)		
	Completely supraportal			9 (7.3 %)	
	Partially supraportal			15 (12.1 %)	
		A6a of infraportal course and A6bc7 of supraportal course			11 (8.9 %)
		A6 of infraportal course and A7 of supraportal course			4 (3.2 %)
Unclear form (because of cancer invasion)			6 (4.8 %)		

*SA/SSA* segmental or subsegmental arteries, *RPV* right portal vein, *A6 and 7* segmental artery supplying segment VI and VII, respectively, *A6a* subsegmental artery suppling ventral segment VI, *A6bc7* subsegmental arteries suppling lateral and dorsal segment VI and segment VII

### Site of SA/SSA bifurcation (extrahepatic/intrahepatic type)

Extrahepatic bifurcation of the SA/SSA branches was revealed in 82 patients (66.1 %), consisting of 39 with the typical form and all 43 with the atypical form (Table 4 and Figs. 3 and 4). The remaining 36 patients with the typical form (29.0 %) were the intrahepatic type.

**Table 4:** The bifurcation site of the SA/SSA (intrahepatic/extrahepatic type)

Intrahepatic type		36 (29.0 %)	
Extrahepatic type		82 (66.1 %)	
	Branched off before main bifurcation of Aant and Apost (atypical form)		43
	Branched off after main bifurcation of Aant and Apost (typical form)		39
Unclear (because of cancer invasion)		6 (4.9 %)	

*SA/SSA* segmental or subsegmental arteries, *Aant and Apost* anterior and posterior hepatic artery

## Discussion

Lymph node dissection in RS is essential in left-sided hepatectomy for hilar cholangiocarcinoma. In recent years, left-sided hepatectomy with concomitant resection and reconstruction of RHA may offer a better chance of long-term survival in patients with the Bismuth type IIIb cholangiocarcinoma involving RHA [1–3]. However, small SA/SSA, which early bifurcate from the RHA and separately enter the hepatic parenchyma, are often found in RS during lymph node dissection. Therefore, a detail preoperative examination of these vascular bifurcation patterns around RS is more crucial in en block arterial resection with left-sided hepatectomy for hilar cholangiocarcinoma to avoid postoperative complications, such as hepatic infarction and subsequent hepatic failure. The present study clarified the various anatomical patterns of SA/SSA around the RS from three viewpoints, bifurcation patterns of the SA/SSA (typical or atypical form), course of the posterior SA/SSA for the RPV (supraportal or infraportal course) and bifurcation site of the SA/SSA (extrahepatic or intrahepatic bifurcations).

The ramification variant of RHA, bifurcated from the superior mesenteric artery or the celiac trunk, has already been reported [5]. On the other hand, there have been few detailed reports on anatomical variants of the SA or SSA around the RS. In this study, atypical SA or SSA (A6 or A6a and so on), which bifurcated independently prior to the main bifurcation of Aant and Apost, were observed in 34.7 % of the hilar cholangiocarcnioma patients. Furthermore, in cases with the atypical form, the early-bifurcated posterior SA/SSA following supraportal course, which is called as “partially supraportal type”, was seen in more than 30 %. In total cohort of our study, the frequency of supraportal posterior branches was 19.5 %, similar to that in a previous report [4]. In Bismuth type IIIb patients with supraportal posterior branches, the risk of intraoperative arterial injury during left-sided hepatectomy is increased. Particularly, during left trisectionectomy for patients with partially supraportal branches, meticulous attention is needed to avoid mistaking partially suprapotal branches (A7 and so on) for the anterior ones. Misidentification of the supraportal posterior branches can result in severe postoperative complications, such as hepatic infarction and hepatic failure [4]. Additionaly, in *en bloc* resection and reconstruction of RHA with cancer invasion, the uninvolved distal arterial branch should be identified around RS before hepatic transection. However, in cases of supraportal Apost, it is difficult to find uninvolved distal Apost. Since supraportal Apost runs behind portal pedicles, distal Apost is usually exposed on the cutting surface during or after hepatic transection, increasing the risk of arterial injury. Therefore, preoperative examination of the bifurcation pattern of SA/SSA and the course of the posterior SA/SSA (supraportal or partially supraportal) using MDCT is mandatory for left-sided hepatectomy, especially left trisectionectomy.

Anatomical variations of an artery, portal vein or bile duct commonly arise around the hepatic hilum before forming Glisson’s triad. Since Glisson’s triad is formed with an intimate fixation in the liver parenchyma, abnormal ramification of these vessels is rare in the liver parenchyma [6]. In this study, the SA/SSA were bifurcated extrahepatically (extrahepatic type) in 82 (66.1 %) of total cohort, all 43 atypical forms and in 39 of 75 typical forms. Even in typical forms, the extrahepatic bifurcation of SA/SSA was observed in 52 %. There is a limitation in our classification of the two types, the extrahepatic or intrahepatic types, due to the difficulty in identifying the starting point of Glisson’s sheath using MDCT. In other words, these types do not necessarily correspond to an anatomical boundary between the outside and inside of the liver. However, based on the CT criteria of the present study, a bifurcation of the SA/SSA determined to be the extrahepatic type is usually exposed during lymph node dissection for RS. Furthermore, even in some patients classified as the intrahepatic type, the bifurcation of the SA/SSA might be exposed at the extrahepatic area in cases of left trisectionectomy or soft hepatic parenchyma, enabling the RS to be opened easily. Our results showed that the extrahepatic type occurred with a high incidence (66.1 %).

In this study, arterial variation around the RS evaluated with preoperative MDCT was not confirmed intraoperatively in all cases because we included cases of right-sided hepatectomy. However, imagining of the vascular 3D structure has been dramatically improved due to advances in the spatial resolution of volume-rendered multidetector CT angiography. Consequently, homology between the preoperative anatomical imaging and intraoperative findings has been reported [4]. Meanwhile, in recent years, the advent of imaging analysis software such as SYNAPSE Vincent® (Fuji Film) has enabled 3D visualization of the vascular structure and liver volumetry of perfused regions, and is expected to be applied for intraoperative navigation [7, 8]. Nevertheless, there are limitations in the automated vascular analysis of these kinds of software. A thin and complex vessel configuration is not necessarily delineated by the software, such as branches of the SA/SSA in RS. Surgical procedures in which injury to the SA/SSA has a critical impact on the residual hepatic function still need a conventional detailed investigation using MDCT to obtain 3D imaging of the anatomical structure.

## Conclusion

Extrahepatic bifurcation of the SA/SSA from RHA is relatively common (66.1 %). In particular, more than 30 % of the early-branched posterior SA/SSA from RHA (atypical form) often follow a supraportal course. Although the anatomical variation of arterial branches around the RS is complex, detailed preoperative knowledge of the anatomy, including SA/SSA, is crucial for left-sided hepatectomy for hilar cholangiocarcinoma.

## Abbreviations

3D images, three-dimensional images; Aant, anterior hepatic artery; Apost, posterior hepatic artery; MDCT, multidetector-row computed tomography; MPR, multi planar reformation; RHA, right hepatic artery; RPV, right portal vein; RS, Rouviere’s sulcus; SA, segmental artery; SSA, subsegmental arteries
